# Supplementary material for: Accounting for heading date gene effects allows detection of small-effect QTL associated with resistance to Septoria nodorum blotch in wheat
Source: PLoS One. 2022 May 19;17(5):e0268546. doi: 10.1371/journal.pone.0268546 (PMC9119491; doi:10.1371/journal.pone.0268546)
Supplement: S2 Fig — Dashed vertical and horizontal lines indicate the GADH population mean for each trait. (PDF) [file pone.0268546.s002.pdf]

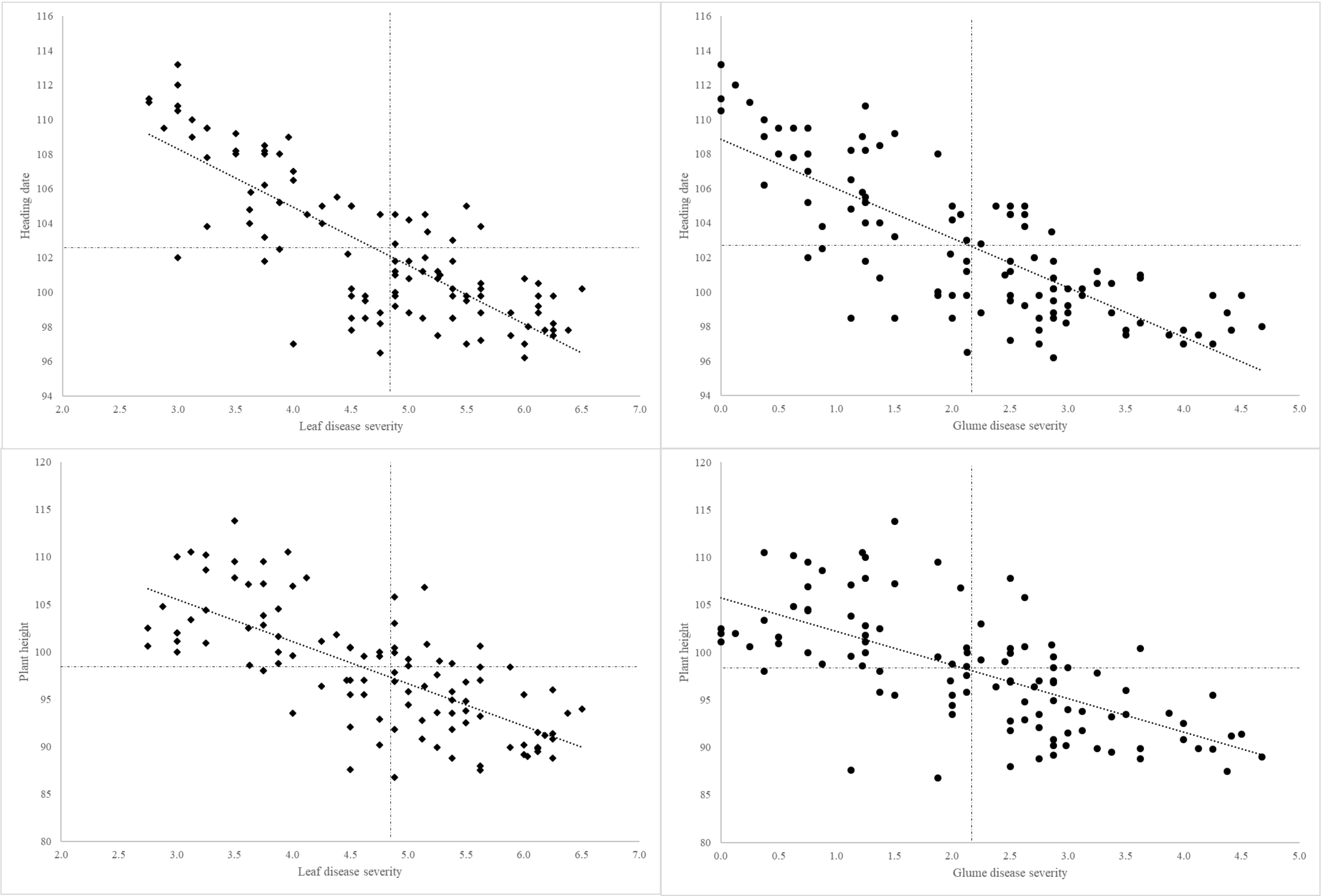

S2 Fig. Scatter plots of combined-year plant height and heading date vs. combined-year leaf and glume disease severity in the GADH population. Dashed vertical and horizontal lines indicate the GADH population mean for each trait.
