# Supplementary material for: Accounting for heading date gene effects allows detection of small-effect QTL associated with resistance to Septoria nodorum blotch in wheat
Source: PLoS One. 2022 May 19;17(5):e0268546. doi: 10.1371/journal.pone.0268546 (PMC9119491; doi:10.1371/journal.pone.0268546)
Supplement: S3 Fig — The vertical axis reflects genetic distance (cM = centiMorgans). The horizontal axis represents the linkage groups assigned to all 21 wheat chromosomes. (PDF) [file pone.0268546.s003.pdf]

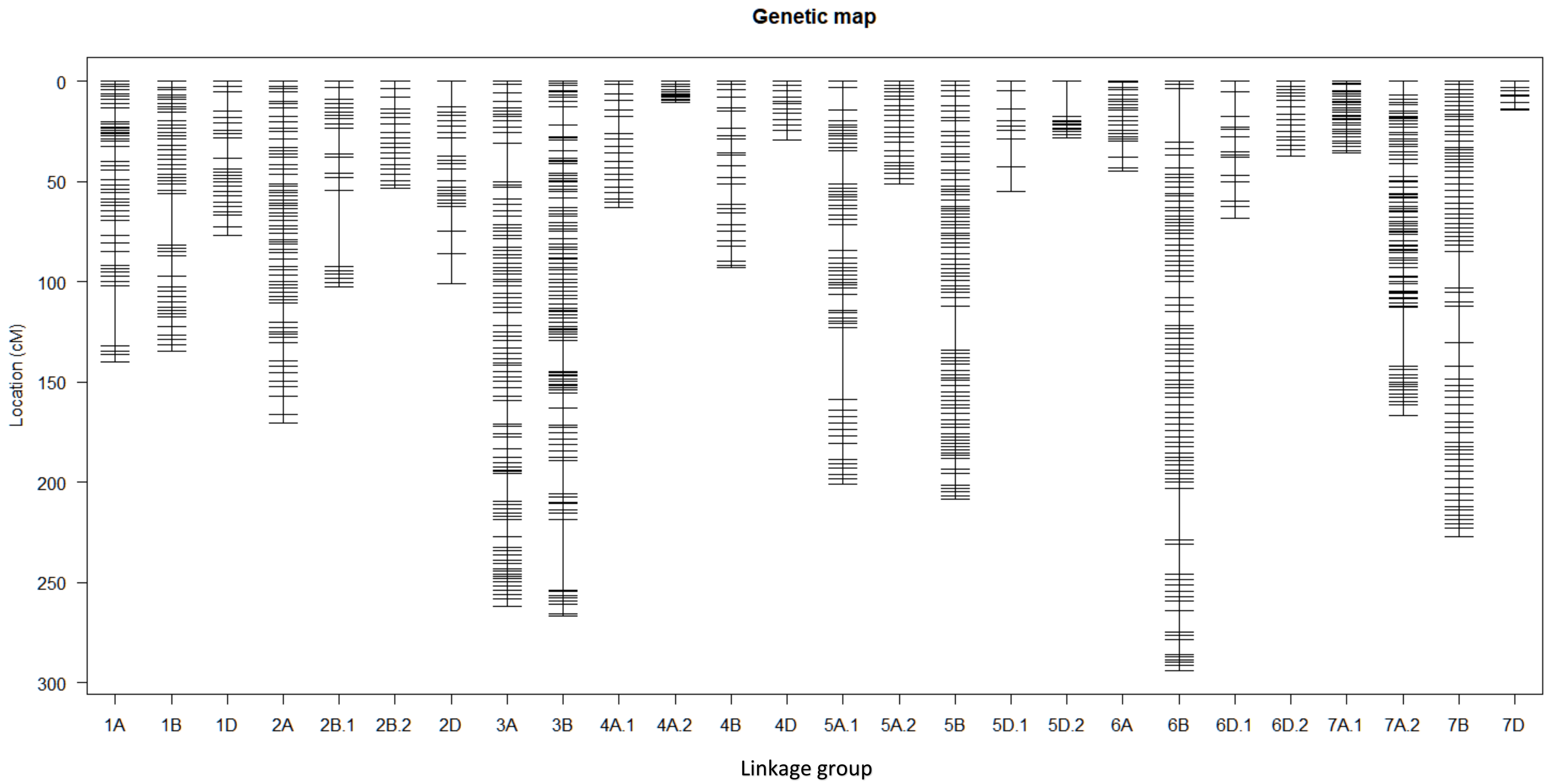

S3 Fig. Genotyping by sequencing single nucleotide polymorphism (GBS-SNP) marker distribution for the 26 linkage groups of the GADH population derived from the AGS2033 x GA03185-12LE29 cross. The vertical axis reflects genetic distance (cM = centiMorgans). The horizontal axis represents the linkage groups assigned to all 21 wheat chromosomes.
