# Supplementary material for: Accounting for heading date gene effects allows detection of small-effect QTL associated with resistance to Septoria nodorum blotch in wheat
Source: PLoS One. 2022 May 19;17(5):e0268546. doi: 10.1371/journal.pone.0268546 (PMC9119491; doi:10.1371/journal.pone.0268546)
Supplement: S1 Table — Abbreviation: Chr = chromosome. (PDF) [file pone.0268546.s004.pdf]

Supporting information

S1 Table. KASP assays for major genes and Robertsonian translocation used in this study.

Locus	Chr	Marker name	Primer name	Sequence	Reference
t1RS·1AL	1A	IWA8035	IWA8035_AL1	GAAGGTGACCAAGTTCATGCTCTTGAGGTCATGACAGAATCTGC	This manuscript
			IWA8035_AL2	GAAGGTCGGAGTCAACGGATTCTCTTGAGGTCATGACAGAATCTGT	
			IWA8035_C1	CAAGAAACCCTGATCCTTCAAACAAGATA	
t1RS·1BL	1B	IWA6110	IWA6110_AL1	GAAGGTGACCAAGTTCATGCTGGAGCAGGTCCAGATCGCG	This manuscript
			IWA6110_AL2	GAAGGTCGGAGTCAACGGATTCGGAGCAGGTCCAGATCGCA	
			IWA6110_C1	GAAGCTCCGGTAGATGGAGGCTA	
PPD-A1	2A	Ppd-A1prodel	Ppd-A1prodel_AL1	GAAGGTGACCAAGTTCATGCTTTTCGGTGTTTGACTTCAGGCG	Nishida et al. 2013; Guedira et al. 2016
			Ppd-A1prodel_AL2	GAAGGTCGGAGTCAACGGATTGCGGCGAGCCGGTTAATCG	
			Ppd-A1prodel_C1	GTGGCGTACTCCCTCCGTTTCTT	
PPD-D1	2D	wMAS000024	TaPpdDD001RI	GAAGGTGACCAAGTTCATGCTCAAGGAAGTATGAGCAGCGGTT	www.cerealsdb.uk.net/cerealgenomics/CerealsDB/kasp_download.php?URL=
			TaPpdDD001RD	GAAGGTCGGAGTCAACGGATTAAGAGGAAACATGTTGGGGTCC	
			TaPpdDD001FL	GCCTCCCACTACACTGGGC	
VRN-B1	5B	TaVrn-B1_1752	vrn-B1_AGS2K_A_ALA	GAAGGTCGGAGTCAACGGATTGGAATGACCGCTGCTTAGTAAATATA	Guedira et al. 2014
			vrn-B1_AGS2K_A_ALC	GAAGGTGACCAAGTTCATGCTGGAATGACCGCTGCTTAGTAAATATC	
			vrn-B1_AGS2K_A_C2	GATTTAGCACCTCAACATACAGGTCT	

Abbreviation: Chr = chromosome.
