# Supplementary material for: Accounting for heading date gene effects allows detection of small-effect QTL associated with resistance to Septoria nodorum blotch in wheat
Source: PLoS One. 2022 May 19;17(5):e0268546. doi: 10.1371/journal.pone.0268546 (PMC9119491; doi:10.1371/journal.pone.0268546)
Supplement: S2 Table — Abbreviations: PH20 = plant height 2020 (cm); PH21 = plant height 2021 (cm); PH = combined-year plant height (cm); HD20 = heading date 2020 (days); HD21 = heading date 2021 (days); HD = combined-year heading date (days); LS18 = leaf severity 2018; LS19 = leaf severity 2019; LS20 = leaf severity 2020; LS21 = leaf severity 2021; LS, combined-year leaf severity; GS18 = glume severity 2018; GS19 = glume severity 2019; GS20 = glume severity 2020; GS21 = glume severity 2021; GS = combined-year glume severity. ** P-value is less than 0.01. (PDF) [file pone.0268546.s005.pdf]

S2 Table. Pearson's correlation coefficients of heading date, plant height, leaf and glume disease severity in the GADH population.

Trait	HD21	HD	PH20	PH21	PH	LS18	LS19	LS20	LS21	LS	GS18	GS19	GS20	GS21	GS	Mean ± S.E.
HD20	0.78**	0.91**	0.73**	0.51**	0.68**	-0.60**	-0.75**	-0.64**	-0.73**	-0.79**	-0.71**	-0.76**	-0.33**	-0.39**	-0.70**	100.2 ± 0.3
HD21	-	0.96**	0.71**	0.46**	0.64**	-0.51**	-0.70**	-0.61**	-0.76**	-0.73**	-0.73**	-0.75**	-0.38**	-0.52**	-0.75**	105.4 ± 0.5
HD		-	0.76**	0.51**	0.69**	-0.58**	-0.75**	-0.66**	-0.79**	-0.80**	-0.76**	-0.80**	-0.37**	-0.49**	-0.77**	102.7 ± 0.4
PH20			-	0.70**	0.93**	-0.58**	-0.72**	-0.61**	-0.66**	-0.75**	-0.70**	-0.75**	-0.37**	-0.36**	-0.71**	91.5 ± 0.7
PH21				-	0.91**	-0.42**	-0.49**	-0.49**	-0.55**	-0.52**	-0.50**	-0.51**	-0.15	-0.24**	-0.43**	105.0 ± 0.6
PH					-	-0.54**	-0.66**	-0.60**	-0.65**	-0.69**	-0.66**	-0.69**	-0.28**	-0.33**	-0.62**	98.2 ± 0.6
LS18						-	0.63**	0.62**	0.56**	0.83**	0.79**	0.62**	0.33**	0.49**	0.69**	5.8 ± 0.1
LS19							-	0.67**	0.72**	0.83**	0.73**	0.85**	0.30**	0.42**	0.75**	4.8 ± 0.1
LS20								-	0.64**	0.81**	0.70**	0.66**	0.36**	0.40**	0.68**	4.8 ± 0.1
LS21									-	0.79**	0.73**	0.72**	0.29**	0.51**	0.71**	4.3 ± 0.1
LS										-	0.83**	0.82**	0.42**	0.55**	0.81**	4.8 ± 0.1
GS18											-	0.80**	0.55**	0.54**	0.91**	3.6 ± 0.2
GS19												-	0.44**	0.50**	0.87**	2.3 ± 0.2
GS20													-	0.23**	0.63**	2.4 ± 0.1
GS21														-	0.65**	1.3 ± 0.1
GS															-	2.2 ± 0.1

Abbreviations: PH20 = plant height 2020 (cm); PH21 = plant height 2021 (cm); PH = combined-year plant height (cm); HD20 = heading date 2020 (days); HD21 = heading date 2021 (days); HD = combined-year heading date (days); LS18 = leaf severity 2018; LS19 = leaf severity 2019; LS20 = leaf severity 2020; LS21 = leaf severity 2021; LS, combined-year leaf severity; GS18 = glume severity 2018;

GS19 = glume severity 2019; GS20 = glume severity 2020; GS21 = glume severity 2021; GS = combined-year glume severity. ** P-value is less than 0.01.
