# Supplementary material for: Accounting for heading date gene effects allows detection of small-effect QTL associated with resistance to Septoria nodorum blotch in wheat
Source: PLoS One. 2022 May 19;17(5):e0268546. doi: 10.1371/journal.pone.0268546 (PMC9119491; doi:10.1371/journal.pone.0268546)
Supplement: S3 Table — Abbreviations: LG = linkage group; cM = centiMorgans; Chr = chromosome; Ave. = average; Max. = maximum; bp = base pairs. (PDF) [file pone.0268546.s006.pdf]

S3 Table. Summary statistics of the genetic linkage map of the GADH population

LG name	Markers	Chr. length (cM)	Ave. spacing (cM)	Max. spacing	Start (bp)	End (bp)
1A	128	117.4	0.9	30.7	251,589,365	597,303,405
1B	146	134.9	0.9	25.6	86,644,505	684,201,943
1D	45	76.7	1.7	10.2	6,738,171	395,425,520
2A	192	170.8	0.9	9.8	35,346,258	774,605,283
2B.1	83	102.8	1.3	38.0	1,940,728	723,217,809
2B.2	48	53.5	1.1	6.0	778,717,630	809,216,419
2D	60	101.0	1.7	14.9	2,702,884	53,583,711
3A	226	261.8	1.2	19.5	12,071,872	748,387,035
3B	198	266.8	1.4	35.1	15,344,850	848,034,513
4A.1	33	62.8	2.0	8.8	565,502,564	617,933,868
4A.2	27	10.5	0.4	1.6	731,559,360	751,727,026
4B	58	93.1	1.6	10.3	18,741,040	661,391,942
4D	23	29.4	1.3	4.8	187,708,264	489,526,775
5A.1	113	201.0	1.8	36.0	2,217,209	587,097,661
5A.2	42	51.5	1.3	4.3	676,479,423	700,614,975
5B	260	208.5	0.8	21.9	217,430	642,176,075
5D.1	11	54.8	5.5	13.7	426,089,393	479,870,959
5D.2	20	28.3	1.5	17.8	533,477,919	554,119,625
6A	41	44.9	1.1	7.6	12,087,561	35,367,474
6B	293	293.9	1.0	26.8	819,075	730,337,799
6D.1	21	68.2	3.4	12.6	11,520,393	344,587,184
6D.2	41	37.3	0.9	4.1	476,389,478	492,658,594
7A.1	69	35.8	0.5	3.1	1,458,996	33,043,068
7A.2	154	167.0	1.1	29.3	87,425,373	710,051,633
7B	314	227.3	0.7	18.6	36,177,089	763,411,793
7D	13	14.5	1.2	3.2	108,220,393	401,394,268
Overall	2,659	2,914.3	1.1	38.0	-	-

Abbreviations: LG = linkage group; cM = centiMorgans; Chr = chromosome; Ave. = average; Max. = maximum; bp = base pairs.
