# Supplementary material for: Accounting for heading date gene effects allows detection of small-effect QTL associated with resistance to Septoria nodorum blotch in wheat
Source: PLoS One. 2022 May 19;17(5):e0268546. doi: 10.1371/journal.pone.0268546 (PMC9119491; doi:10.1371/journal.pone.0268546)
Supplement: S4 Table — Abbreviations: CIM = composite interval mapping; SIM = standard interval mapping; QTL = quantitative trait loci; LG = linkage group; cM = centimorgan; HD = heading day (days); PH = plant height (cm); HD20 = heading date 2020; HD21 = heading date 2021; PH20 = plant height 2020; PH21 = plant height 2021; LOD = logarithm of the odds at 0.05 level of probability, obtained through a 5,000-iteration permutation test; PVE = percentage of phenotypic variance explained by the QTL. aEstimated allele effect reported in terms of the AGS2033 parent. The estimated allele effect unit corresponds to each trait unit. c = covariate. (PDF) [file pone.0268546.s007.pdf]

**S4 Table. Within-year QTL associated with heading date and plant height in the GADH population using the CIM and SIM with major genes as covariates.**

| CIM | | | | | | | | |
|---|---|---|---|---|---|---|---|---|
| Trait | QTL | LG | cM | Confidence interval (cM) | LOD | *P*-value | PVE (%) | Allele effect[a] |
| HD20 | *Ppd-A1* | 2A | 13 | 10-16 | 6.7 | 0.0000 | 23.2 | 1.7 |
| | *Ppd-D1* | 2D | 86 | 83-89 | 10.5 | 0.0000 | 34.0 | 2.0 |
| | *Qncb.hd-5A* | 5A.1 | 191 | 186-191 | 4.8 | 0.0016 | 7.9 | 1.0 |
| | *Qncb.hd-5B* | 5B | 148 | 87-173 | 4.5 | 0.0024 | 12.2 | -1.2 |
| HD21 | *Ppd-A1* | 2A | 13 | 10-16 | 6.8 | 0.0002 | 20.2 | 2.6 |
| | *Ppd-D1* | 2D | 86 | 83-89 | 14.7 | 0.0000 | 42.5 | 3.7 |
| | *Qncb.hd-5B* | 5B | 173 | 170-176 | 13.7 | 0.0000 | 36.1 | -3.3 |
| PH20 | *Ppd-A1* | 2A | 13 | 10-16 | 5.6 | 0.0000 | 19.3 | 3.2 |
| | *Qncb.ph-2D* | 2D | 88 | 83-89 | 9.8 | 0.0000 | 47.1 | 5.1 |
| | *Qncb.ph-5A* | 5A.1 | 189 | 151-201 | 3.5 | 0.0170 | 4.0 | 1.5 |
| PH21 | *Qncb.ph-2D* | 2D | 88 | 50-89 | 3.3 | 0.0282 | 30.9 | 3.8 |
| SIM with covariates | | | | | | | | |
| HD20 | *Qncb.hd-5A*[c] | 5A.1 | 201 | 186-201 | 6.6 | 0.0000 | 8.4 | 1.0 |
| HD21 | *Qncb.hd-5B*[c] | 5B | 80 | 22-105 | 3.3 | 0.0340 | 13.1 | -2.0 |
| PH20 | *Qncb.ph-2D*[c] | 2D | 40 | 29-61 | 4.1 | 0.0042 | 24.0 | 3.5 |
| | *Qncb.ph-5A*[c] | 5A.1 | 181 | 146-201 | 3.1 | 0.0414 | 4.4 | 1.5 |

Abbreviations: CIM = composite interval mapping; SIM = standard interval mapping; QTL = quantitative trait loci; LG = linkage group; cM = centimorgan; HD = heading day (days); PH = plant height (cm); HD20 = heading date 2020; HD21 = heading date 2021; PH20 = plant height 2020; PH21 = plant height 2021; LOD = logarithm of the odds at 0.05 level of probability, obtained through a 5,000-iteration permutation test; PVE = percentage of phenotypic variance explained by the QTL. [a]Estimated allele effect reported in terms of the AGS2033 parent. The estimated allele effect unit corresponds to each trait unit. [c] = covariate.
