# Supplementary material for: Accounting for heading date gene effects allows detection of small-effect QTL associated with resistance to Septoria nodorum blotch in wheat
Source: PLoS One. 2022 May 19;17(5):e0268546. doi: 10.1371/journal.pone.0268546 (PMC9119491; doi:10.1371/journal.pone.0268546)
Supplement: S5 Table — Abbreviations: CIM = composite interval mapping; SIM = standard interval mapping; QTL = quantitative trait loci; LG = linkage group; cM = centimorgan; LS = leaf severity (1–9 scale); GS = glume severity (0–9 scale); LS18 = leaf severity 2018; LS19 = leaf severity 2019; LS20 = leaf severity 2020; LS21 = leaf severity 2021; GS18 = glume severity 2018; GS19 = glume severity 2019; GS20 = glume severity 2020; GS21 = glume severity 2021; LOD = logarithm of the odds at 0.05 level of probability, obtained through a 5,000-iteration permutation test; PVE = percentage of phenotypic variance explained by the QTL. aEstimated allele effect reported in terms of the AGS2033 parent. The estimated allele effect unit corresponds to each trait unit. c = covariate. (PDF) [file pone.0268546.s008.pdf]

S5 Table. Within year quantitative trait loci (QTL) associated with leaf disease severity (LS) and glume disease severity (GS) in the GADH population using the CIM and SIM with major genes as covariates.

Trait	QTL	LG	cM	Confidence interval (cM)	LOD	*P*-value	PVE (%)	Allele effect[a]
CIM								
LS18	*Qncb.snl-1A*	1A	24	21-27	4.5	0.0042	12.2	0.5
	Ppd-A1	2A	13	10-16	6.9	0.0002	27.8	-0.7
	Ppd-D1	2D	86	83-89	5.5	0.0012	19.4	-0.6
LS19	*Ppd-A1*	2A	13	10-16	6.3	0.0000	20.1	-0.49
	Qncb.snl-2D	2D	88	83-89	13.2	0.0000	41.4	-0.7
	Qncb.snl-5A	5A.1	196	193-199	6.0	0.0000	9.6	-0.3
	Qncb.snl-5B	5B	148	143-184	3.5	0.0212	8.9	0.3
	Qncb.snl-6B	6B	49	25-174	5.1	0.0010	13.7	0.4
LS20	*Qncb.snl-1A*	1A	4	2-6	3.9	0.0102	12.0	0.4
	Qncb.snl-2D	2D	88	84-88	8.4	0.0000	25.7	-0.6
LS21	*Qncb.snl-2A*	2A	25	23-27	5.8	0.0000	18.3	-0.5
	Ppd-D1	2D	86	84-88	11.7	0.0000	36.9	-0.7
	Qncb.snl-5B	5B	176	174-178	7.5	0.0000	22.9	0.6
GS18	*Qncb.sng-1A*	1A	41	38-44	6.3	0.0000	17.8	0.9
	Qncb.sng-2A	2A	26	23-28	7.9	0.0000	27.7	-1.2
	Qncb.sng-2D	2D	88	83-89	14.2	0.0000	35.7	-1.3
	Qncb.sng-5B	5B	148	138-184	3.6	0.0170	14.7	0.8
GS19	*Ppd-A1*	2A	13	10-16	9.7	0.0000	29.6	-0.8
	Qncb.sng-2D	2D	85	83-89	11.4	0.0000	40.9	-1.0
	Qncb.sng-5B	5B	151	149-155	3.5	0.0234	13.6	0.6
GS20	*Qncb.sng-1A*	1A	41	38-44	5.3	0.0004	20.4	0.4
GS21	*Qncb.sng-2A*	2A	31	30-36	5.2	0.0008	18.2	-0.5
SIM with covariates								
LS18	*Qncb.snl-1A*[c]	1A	24	0-39	4.2	0.0058	12.2	0.5
LS19	*Qncb.snl-5A*[c]	5A.1	201	184-201	6.7	0.0000	9.7	-0.3
	Qncb.snl-6B[c]	6B	76	28-120	5.7	0.0000	13.5	0.4
LS20	*Qncb.snl-1A*[c]	1A	4	0-77	4.7	0.0030	12.0	0.4
	Qncb.snl-1B[c]	1B	11	0-48	4.1	0.0084	11.8	0.4
	Qncb.snl-6B[c]	6B	94	38-174	3.5	0.0246	11.7	0.4
GS18	*Qncb.sng-1A*[c]	1A	24	18-42	8.9	0.0000	14.5	0.8
	Qncb.sng-5B[c]	5B	154	152-155	3.2	0.0388	15.4	0.8
GS20	*Qncb.sng-1A*[c]	1A	36	18-45	5.9	0.0000	19.0	0.4
	Qncb.sng-1B[c]	1B	46	0-70	3.7	0.0150	11.1	0.3

Abbreviations: CIM = composite interval mapping; SIM = standard interval mapping; QTL = quantitative trait loci; LG = linkage group; cM = centimorgan; LS = leaf severity (1-9 scale); GS = glume severity (0-9 scale); LS18 = leaf severity 2018; LS19 = leaf severity 2019; LS20 = leaf severity 2020; LS21 = leaf severity 2021; GS18 = glume severity 2018; GS19 = glume severity 2019; GS20 = glume severity 2020; GS21 = glume severity 2021; LOD = logarithm of the odds at 0.05 level of probability, obtained through a 5,000-iteration permutation test; PVE = percentage of phenotypic variance explained by the QTL. [a]Estimated allele effect reported in terms of the AGS2033 parent. The estimated allele effect unit corresponds to each trait unit. [c] = covariate.
