# Supplementary material for: Accounting for heading date gene effects allows detection of small-effect QTL associated with resistance to Septoria nodorum blotch in wheat
Source: PLoS One. 2022 May 19;17(5):e0268546. doi: 10.1371/journal.pone.0268546 (PMC9119491; doi:10.1371/journal.pone.0268546)
Supplement: Attachment — Submitted filename: Response_to_Reviewers.docx [file pone.0268546.s010.docx]

**Response to reviewers**

Thank you very much for the opportunity to submit a revised version of the manuscript. Please find point-to-point responses to the reviewer’s comments below. We thank the editor and the reviewers for valuable input and hope that you will find the responses acceptable.

**PONE-D-21-34614R1**

Overall, our study presents a simple and robust approach not currently reported in the literature to map resistance quantitative trait loci (QTL) to leaf and glume blotch. This approach uses the power of predictive markers associated with developmental traits to control for pleiotropic effects. We revealed SNB QTL on 1A, 1B, and 6B linkage groups. Although we are limited in our ability to draw conclusions about the relationship of these markers with previously reported QTL, the small-effect QTL detected in this study did not overlap with QTL associated with morphological and developmental traits, and thus are sources of resistance to SNB.

**Journal Requirements:**

**______________________________________________________________________________**

*Please review your reference list to ensure that it is complete and correct. If you have cited papers that have been retracted, please include the rationale for doing so in the manuscript text, or remove these references and replace them with relevant current references. Any changes to the reference list should be mentioned in the rebuttal letter that accompanies your revised manuscript. If you need to cite a retracted article, indicate the article’s retracted status in the References list and also include a citation and full reference for the retraction notice.*

**Response:**

All references listed are complete and correct. We have not cited retracted articles. Thank you.

**Reviewers' comments:**

**______________________________________________________________________________**

**Comments to the Author**

*1. If the authors have adequately addressed your comments raised in a previous round of review and you feel that this manuscript is now acceptable for publication, you may indicate that here to bypass the “Comments to the Author” section, enter your conflict of interest statement in the “Confidential to Editor” section, and submit your "Accept" recommendation.*

Reviewer #1: All comments have been addressed.

Reviewer #2: (No Response).

**______________________________________________________________________________**

*2. Is the manuscript technically sound, and do the data support the conclusions?

The manuscript must describe a technically sound piece of scientific research with data that supports the conclusions. Experiments must have been conducted rigorously, with appropriate controls, replication, and sample sizes. The conclusions must be drawn appropriately based on the data presented*.

Reviewer #1: Yes

Reviewer #2: Partly

**______________________________________________________________________________**

*3. Has the statistical analysis been performed appropriately and rigorously?*

Reviewer #1: Yes

Reviewer #2: Yes

**______________________________________________________________________________**

*4. Have the authors made all data underlying the findings in their manuscript fully available?

The* [*PLOS Data policy*](http://www.plosone.org/static/policies.action#sharing) *requires authors to make all data underlying the findings described in their manuscript fully available without restriction, with rare exception (please refer to the Data Availability Statement in the manuscript PDF file). The data should be provided as part of the manuscript or its supporting information, or deposited to a public repository. For example, in addition to summary statistics, the data points behind means, medians and variance measures should be available. If there are restrictions on publicly sharing data—e.g. participant privacy or use of data from a third party—those must be specified.*

Reviewer #1: Yes

Reviewer #2: Yes

**______________________________________________________________________________**

*5. Is the manuscript presented in an intelligible fashion and written in standard English?

PLOS ONE does not copyedit accepted manuscripts, so the language in submitted articles must be clear, correct, and unambiguous. Any typographical or grammatical errors should be corrected at revision, so please note any specific errors here.*

Reviewer #1: Yes

Reviewer #2: Yes

**______________________________________________________________________________**

*6. Review Comments to the Author

Please use the space provided to explain your answers to the questions above. You may also include additional comments for the author, including concerns about dual publication, research ethics, or publication ethics. (Please upload your review as an attachment if it exceeds 20,000 characters)*

*Reviewer #1: (No Response)*

*Reviewer #2: This manuscript reports the identification of resistance QTL to leaf and glume Septoria nodorum blotch (SNB) using a double haploid (DH) population derived from the cross between the moderately susceptible cultivar AGS2033 and the resistant breeding line GA03185-12LE29. And the authors identified major heading date (HD) and plant height (PH) variants on chromosomes 2A and 2D, co-located with SNB loci. Meanwhile, five minor QTL associated with adult plant resistance to SNB leaf and glume blotch were detected on 1A, 1B, and 6B linkage groups. The paper is written in standard English and SNB is a major disease affecting wheat. However, there are several results the authors should be carefully made:*

*1. The rise of SNB is similar to the yellow rust, and the temperature and the humidity had significant effect to the disease. As a result, the growth period may be one of the main reasons of the disease. Based on this, the phenotype identified in the manuscript may not accurate. As we detect the yellow rust, it was detected in several time, e.g. seedling stage and adult plant stage. Is it accurate to identify at only one time?*

**Response:**

We appreciate the reviewer’s concern. The latent period of SNB is usually longer than that of the YR epidemics with which the reviewer is familiar. Thus, the rate of YR disease development is typically much higher than the rate of SNB development. It is common to rate SNB epidemics as disease severity (DS, a single point in time) rather than area under disease progress curve (AUDPC, integrated over time). This reflects that SNB typically only causes economic loss if it heavily affects flag leaves and glumes, while early establishment of YR can cause severe economic loss through stunting and loss of photosynthate for grain fill. No changes were made in response to this comment.

*2. The authors use flowering time genes as covariates to detect SNB QTL. Is this suitable? The relationship of flowering time and the SNB is direct? And flowering time is the component of SNB?*

**Response:**

It was suitable to investigate the relationship between QTL for SNB reaction and determinants of maturity; i.e., to determine whether SNB response was simply due to heading date. In fact, QTL for SNB reaction co-located with known determinants of heading date were identified during preliminary analysis of the first two years of data. This led to an assessment of heading date and plant height in the disease nursery during 2020 and 2021.

This was already explained in the several places:

Page 7 & lines 142-145: “Preliminary QTL analysis of the 2018 and 2019 data identified SNB QTL coinciding with markers for flowering time genes. Days to heading (HD) was then assessed in the 2020 and 2021 experiments…..”

Page 21 & lines 433-438: “Once the flowering loci were taken into account, our QTL analysis did not locate additional QTL for SNB resistance on 2DS but did locate a plant height locus on the chromosome arm. This suggests that disease resistance associated with plant height was due primarily to pleiotropic effects of HD on both PH and disease. In our nursery, irrigation was applied specifically to reduce escapes due to tallness by regularly splashing inoculum up the canopy.”

The genotypic data for the underlying causal polymorphisms in the heading date genes explained the majority of the HD variation in the population in both years, as noted at various places in the results (ie. Table 2) and in the discussion on page 22 & lines 462-464:

“The use of major genes as covariates in a SIM approach was justified as *Ppd-A1*, *Ppd-D1* and *Vrn-B1* accounted for at least two-thirds of the variation in HD and PH in 2020 and 2021, and likely played a large role in these traits in Raleigh during 2018 and 2019.”

No changes were made in response to this comment.

*3. “Each genotype was planted in a single 1.3-meter row, with 3 g of seed per row except in a few cases where seed availability was lower”. “3 g of seed per 1.3-meter” that means there may -70 seeds per 1.3 meter? Is this density too high for plant growth?*

**Response:**

That is a good estimate of the number of seeds per row, and it produces an appropriate density of wheat plants in the North Carolina environments where this experiment was conducted. No changes were made in response to this comment.
